# Induction by anti-thymocyte globulins in kidney transplantation: a review of the literature and current usage

**DOI:** 10.12860/jnp.2015.21

**Published:** 2015-10-01

**Authors:** Paolo Malvezzi, Thomas Jouve, Lionel Rostaing

**Affiliations:** ^1^Clinique de Néphrologie, University Hospital, Grenoble, France; ^2^Department of Nephrology, Dialysis, and Organ Transplantation, CHU Rangueil, Toulouse University Hospital, Toulouse, France; ^3^INSERM U563, IFR–BMT, CHU Purpan, Toulouse, France; ^4^Université Paul Sabatier, Toulouse, France

**Keywords:** Kidney transplantation, Anti-thymocyte globulins, Acute rejection, Cytomegalovirus, *
De novo *cancer

## Abstract

*Context:* Preventing acute rejection (AR) after kidney transplantation is of utmost importance because an AR can have a negative impact on long-term allograft survival.

*Evidence Acquisition:* Directory of Open Access Journals (DOAJ), Google Scholar, PubMed, EBSCO, and Web of Science have been searched.

*Results:* At the moment this can be done by using rabbit anti-thymocyte globulins (rATGs) as an induction therapy. However, because rATGs are associated with some deleterious side-effects, such as the opportunistic infections cytomegalovirus (CMV) and *de novo* post-transplant cancer, it is very important they are used optimally, i.e., at minimal doses that avoid many side-effects but still retain optimal treatment efficacy. Recent data show that the risk of CMV infection can be minimized using tacrolimus plus everolimus, and not tacrolimus plus mycophenolic acid, as the maintenance immunosuppression. The use of rATG is particularly valuable in; *(a) * sensitized patients; *(b)* in recipients from an expanded-criteria donor, thus enabling the introduction of calcineurin inhibitors at reduced doses; and *(c)* for patients where steroid avoidance is contemplated. However, we also need to consider that rATG may increase the risk of *de novo* cancer, even though recent data indicate this is unlikely and that any risk can be reduced by using mammalian target of rapamycin (mTOR) inhibitors instead of mycophenolic acid combined with low-dose calcineurin inhibitors.

*Conclusions:* Even though rATGs do not improve long-term kidney-allograft survival, they may help reduce calcineurin-inhibitor dosage during the early post-transplant period and minimize the risk of AR.

**Implication for health policy/practice/research/medical education:**

The use of anti-thymocyte globulins (rATGs) is particularly valuable in; *(a)* sensitized patients; *(b)* in recipients from an expanded-criteria donor, thus enabling the introduction of calcineurin inhibitors at reduced doses; and *(c)* for patients where steroid avoidance is contemplated. However, we also need to consider that rATG may increase the risk of *de novo* cancer, even though recent data indicate this is unlikely and that any risk can be reduced by using mammalian target of rapamycin (mTOR) inhibitors instead of mycophenolic acid combined with low-dose calcineurin inhibitors.

## 1. Context

Polyclonal antibodies directed against T lymphocytes (ATG) have been developed since discovering the underlying mechanisms involved in acute cellular rejection. Initially derived from horse serum, these drugs have a potent depleting effect but have been frequently associated with serious adverse effects, such as serum sickness, profound pancytopenia, infection, and cancer.

## 2. Evidence Acquisition

Directory of Open Access Journals (DOAJ), Google Scholar, PubMed, EBSCO and Web of Science were searched with key words relevant to kidney transplantation, anti-thymocyte globulins, acute rejection, cytomegalovirus and *de novo* cancer.

## 3. Results

In the early1980s, rabbit-derived anti-thymocyte globulins (rATG) were licensed for use in kidney transplantation: this preparation had a better tolerance profile and was used initially to treat steroid-resistant rejection. Later on, they were introduced as an induction agent within immunosuppressive protocols, and were frequently used with anti-calcineurin inhibitors, anti-proliferative drugs, and steroids. At present, rATGs are the most widely used induction treatments worldwide: in the US alone they are given to >60% of *de novo* kidney-transplant recipients (1).

Two forms of rATG exist: Thymoglobulin^®^(Sanofi), which is extracted and purified from the serum of pediatric thymic tissue–immunized rabbits, and ATG-Fresenius^®^, which is derived from the serum of immunized rabbits with a human T-cell line (Jurkat cells).

Over the past 30 years, since its first commercialization, its quality, dosing schedules, induction protocols, and concomitant drugs, as well as the patients’ characteristics and the clinicians’ expectations have dramatically changed. In this review we summarize and prioritize the most recent data concerning the use of rATGs in modern induction protocols.

### 3.1. rATG dosing

The most interesting point about rATGs is that T-cell depletion is dose-dependent as are their side-effects.

During the early years of rATG induction, standard doses were between 1 and 1.5 mg/kg/day for 7-10 days (total dose of 10-15 mg/kg) (2-9). These high doses were frequently associated with early and late adverse events, such as profound and prolonged leucopenia, thrombocytopenia, infection, and cancer.

Over time, doses used during rATG-induction regimens have been reduced because evidence shows they are equally effective at lower doses while also being less toxic (10). In one study, in high-risk recipients, a mean total dose of 5.7 mg/kg produced similar outcomes to those that received an average of 10.3 mg/kg (10).

More recent published protocols have compared very low doses of rATGs given to low immunological-risk patients. In one small randomized series (11), two low-dose regimens were compared (total doses of 3.75 vs. 2.25 mg/kg) in non-sensitized patients undergoing steroid withdrawal. Both regimens achieved low biopsy-proven acute-rejection rates (17% vs. 10%) with potentially less opportunistic viral infections in the lower dose group.

These very low-dose protocols show that rATG sparing is feasible and probably useful in the early months post-transplantation and in the setting of steroid avoidance or withdrawal.

The question raised by the use of low-dose protocols is what is the lowest dose that still possesses the best therapeutic effect? A useful insight has been given by a Dutch group who evaluated the effect of different doses of rATG on T cells, B cells, and NK cells (12). At 1.5 mg/kg total dose, T cells and NK cells were still depleted at one week post-transplant, but at one month later their numbers had recovered to baseline levels, while patients receiving 3 mg/kg were still T-cell depleted after one month but levels had returned to baseline values by 1 year later. Prolonged T-cell lymphopenia over the first post-transplant year was found in those that received 6 mg/kg of rATGs. These results infer that, the lower the dose, the more we should expect this treatment to be ineffective and have a lesser potential benefit.

In addition, different dosing regimens may be required for different profiles of patients: i.e., lower doses for low immunological-risk patients and elderly recipients; and higher doses for those with a higher risk and for recipients of an expanded-criteria donor kidney to reduce the toxicity from calcineurin inhibitors given during the early post-transplant period (13).

### 3.2. Different induction strategies

Until recently, induction has been mostly reserved for immunized patients with high titers of anti-HLA alloantibodies or who have undergone repeat transplantations to reduce acute-rejection episodes soon after transplantation. Thus, some studies have explored the use of rATGs in low-risk populations.

### 3.3. rATGs versus no induction

Two short-term randomized trials report that recipients who received a kidney from a deceased donor had a reduced rejection rate when rATG was used as the induction therapy (14,15). A parallel 6-month multicenter European trial included 555 recipients of a diseased-donor kidney. They were randomized into either receiving calcineurin inhibitors (CNI; either tacrolimus or cyclosporine), steroids, azathioprine, plus rATG as the induction therapy (1.25 mg/kg for 10 days), or tacrolimus, azathioprine, plus steroids, without an induction therapy (14). Patients treated with rATG–tacrolimus had the lowest incidence of biopsy-proven acute rejection (BPAR) even though this difference did not reach significance (15.1% vs. 21.2% rATG–cyclosporine and 25.4% tacrolimus–no induction; *P *= ns). However, patient- and graft-survival rates, as well as serum-creatinine levels, were similar in both groups at 6 months post-transplantation. In addition, patients receiving the rATG treatment experienced a significantly higher incidence of leucopenia, thrombocytopenia, serum sickness, fever, and cytomegalovirus (CMV) infection (*P *< 0.05).

Another multicenter, randomized study was conducted in Europe and included 309 *de novo* low-risk immunological kidney-transplant recipients who had received a graft from a deceased donor (15). The kidney recipients received either rATG (n = 151, 1.25 mg/kg for 10 days) followed by a tacrolimus-based triple therapy (i.e., tacrolimus was started on day 9), or received no induction treatment and the tacrolimus-based triple therapy was started immediately following transplantation (n = 158) (15). The incidence of BPAR was significantly lower in the rATG group (15.2% vs. 30.4%; *P *= 0.001). At 12 months, patient- and graft-survival rates, as well as renal function and delayed graft function, were similar between both groups. Adverse events, such as leucopenia, thrombocytopenia, and CMV infections, were more frequently reported in patients who had received an induction therapy.

Although these two trials demonstrated that a rATG-induction therapy can delay the introduction of CNIs and lower the incidence of early-rejection episodes, this was at the expense of reversible thrombocytopenia and leucopenia, and increased infection, particularly CMV.

Moreover, despite significantly decreasing the rate of BPAR, the induction therapy seemed to have no significant impact on one-year patient-survival, or graft function or survival. Therefore low-risk patients treated with a modern immunosuppressive treatment that includes a CNI, an anti-proliferative, and steroids, may not need a rATG-induction regimen.

### 3.4. Early steroid-withdrawal strategies supported by lymphocyte-depleting induction therapies

Another way to profit from the immunosuppressive potential of rATG induction is to rapidly withdraw steroids in specific patients. The focus of this strategy is to reduce the morbidity tied to chronic steroid exposure, such as diabetes, weight gain, hyperlipidemia, and hypertension.

A few prospective randomized trials have explored this possibility and all show that when patients are treated with a maintenance immunosuppressive regimen coupled with CNIs and mycophenolate mofetil, rATG induction allows safe withdrawal of steroids within the first three months (16-19). All trials show either a lower or similar BPAR rate in rATG-treated patients. Some trials have also described a better metabolic profile when steroids were withdrawn (19).

### 3.5. CNI avoidance or CNI sparing with rATG induction

While CNI sparing or the late introduction of CNIs is a widespread and relatively accepted strategy, complete CNI avoidance is still debated.

A few prospective studies show that the late introduction of CNIs is feasible when patients undergo an induction with rATGs, with this strategy being especially useful when there are risk factors for delayed graft function (20,21): i.e., older donor age, long ischemia time, a vascular donor.

CNI avoidance plus a maintenance therapy of mammalian target of rapamycin (mTOR) inhibitors and mycophenolate mofetil have shown excellent one-year results regarding BPAR and patients’ survival (22-24). However, only one study has actually demonstrated lower graft survival in a CNI-free group (22). The question then arises as to what happens in the long run and whether these patients, once the immunosuppressive effect of rATG disappears, develop *de novo* donor-specific alloantibodies (dnDSAs): indeed, m-TOR inhibitor-based immunosuppressive regimens may enhance this phenomenon (25,26).

### 3.6. Delayed graft function and induction therapy

It has been reported that intraoperative administration of anti-thymocyte globulins may minimize the lesions caused by ischemia-reperfusion injury, and the subsequent development of delayed graft function. Hence, a prospective, randomized trial on recipients of a *de novo* kidney transplant from a deceased donor found that, compared to postoperative administration, giving the first dose of rATG intraoperatively was associated with less delayed graft function (14.8% intraoperatively vs. 35.5% postoperatively; *P *< 0.05) and resulted in significantly better renal function by day 14 (serum creatinine 1.81 vs. 2.82 mg/dL; *P *= 0.04) (27). However, these findings have not been reproduced since.

### 3.7. Effect of rATG on long-term patient- and graft-survival rates

What about long-term survival? Only registry data are available to answer this question; unfortunately, there are few results from long-term randomized controlled studies. The European Collaborative Transplant Study, coordinated by Opelz et al, has reported higher 3-year allograft-survival rates in patients receiving a first transplant and who had received an induction therapy of either rATG or IL2RA, when compared to receiving no induction, but this resulted in, at least for rATG, a higher rate of non-Hodgkin’s lymphomas (28). More recent US data from the OPTN registry (29) focused on induction therapies in living-donor transplantation and where the recipients received the more ‘modern’ immunosuppression of tacrolimus, mycophenolate mofetil, and steroids. The results actually showed no benefit to kidney-allograft survival at 5 years after using any type of antibody in the induction therapy, although the non-induction group actually suffered from a greater 5-year mortality.

Finally, very few data have reported on the capacity of rATG induction to reduce dnDSA development and thus the incidence of consequent acute or chronic antibody-mediated rejections. One recent publication showed that rATG was superior to IL2 receptor antagonists in preventing dnDSAs and antibody-mediated rejection in a moderately sensitized population (30). These very limited data strengthen the belief that rATG is not exclusively a T-cell depleting agent, but can probably also alter B-cell function either by disrupting antigen presentation and/or T-cell-to-B-cell crosstalk.

### 3.8. Drawbacks

Since its introduction, rATGs have been associated with all the drawbacks of strong immunosuppressive regimens, i.e., infections and *de novo* cancers.

Infectious complications are common during the post- transplant period but the main risksignificantly associated with rATG treatment is CMV. The more often it affects CMV-seronegative patients receiving a CMV-seropositive kidney, and has been described in almost all clinical trials in which a rATG-induction therapy has been performed. However, in more recent protocols where lower doses of rATG are used, the prevalence of CMV disease is reported as less serious. Various strategies have been adopted to reduce the risk of CMV infection: CMV prophylaxis based on (val) ganciclovir is widely used when treating patients with rATG. More recently, an interesting study showed the efficacy of an original immunosuppressive strategy where rATG induction was associated with low-dose tacrolimus and everolimus. In this study, the authors achieved an extremely low incidence of CMV disease even without CMV prophylaxis (10%) when compared to induction with basiliximab-, tacrolimus-, and mycophenolate mofetil-based immunosuppression (37%) (31).

Cancer, and in particular post-transplant lymphoproliferative disorders (PTLD), has been described to occur more frequently in patients that have received rATG. European (28) and US registry (32) reports show higher incidences of PTLD when T-cell depleting agents are used for AR or as an induction therapy, when compared to no induction at all. But if we look only at rATG use as an induction treatment the data are conflicting between the two registries: the American registry does not show any difference, whereas the European registry shows a worse risk profile for rATG (particularly Thymoglobulin^®^). This apparent difference is probably because of the difference in these two cohorts: in Europe rATG was used in historically earlier periods for induction, i.e., before the 2000s and at higher doses, whereas in the United States, the use of rATG as an induction therapy gained momentum after the year 2000 and now almost 60% of patients receive low-dose rATG as an induction.

A very recent study by Hall et al (33) has confirmed, by comparing the different registries (and thus obtaining more precise data), that rATG use is not associated with lymphoma nor with any solid cancer except for melanoma in the non-Hispanic white population. This finding is reassuring and confirms a recent meta-analysis in which the authors showed how difficult it is to interpret retrospective studies and that the only solid evidence states that: “the lower the dose, the lower the risk” (34).

## 4. Conclusions

rATGs are now an integral part of the transplant clinician’s armament. With time, experience has shown us the dangers of over immunosuppression as rATG dosing protocols have evolved. Although rATG was used at first almost exclusively for induction in high-risk patients or to treat AR, increasing numbers of patients now receive small doses of rATG to reduce exposure to steroids and CNIs.

Unfortunately, good prospective randomized trials are lacking, and are needed to establish whether these low-dose rATG protocols actually benefit patients in the long term, especially when coupled with modern immunosuppressive maintenance regimens of low-dose tacrolimus plus mycophenolic acid. *De novo* DSA development and consequent chronic antibody-mediated rejection may be reduced by rATG treatment, but more prospective data are required.

The most recent studies show that *de novo* cancer rate is not significantly higher in rATG-treated patients compared to patients not receiving an induction therapy, but caution needs to be taken when a high or cumulative dose of this potent immunosuppressor is given.

Finally, as far as infection is concerned, CMV seems to be more frequent: thus, prophylaxis or different antiviral strategies, e.g. by replacing mycophenolic acid by everolimus need to be considered, especially for CMV seronegative recipients with a CMV seropositive donor.

## Authors’ contribution

Primary draft by PM and TJ. Manuscript edit by LR. All authors read and signed the final paper.

## Conflicts of interest

The authors declare no conflict of interest.

## Funding/Support

None.
